# This Volcanologist Peers into “Crystal Balls” to Forecast Eruptions

**DOI:** 10.1021/acscentsci.6c00452

**Published:** 2026-03-25

**Authors:** Jonathan Feakins

## Abstract

Microscopic minerals help Teresa Ubide understand volcanoes—

## Graphical abstract

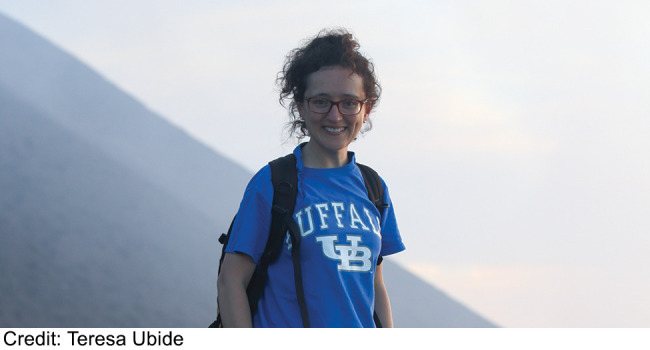

Nearly 1 in 10 people live within 100 km of an active volcano. From Reykjavík, Iceland, to Yogyakarta, Indonesia, communities have accumulated deep awareness of—and respect for—the risks and challenges of living alongside these geologic wonders. The world underneath them, on the other hand, has remained far harder to comprehend.

**Figure:**
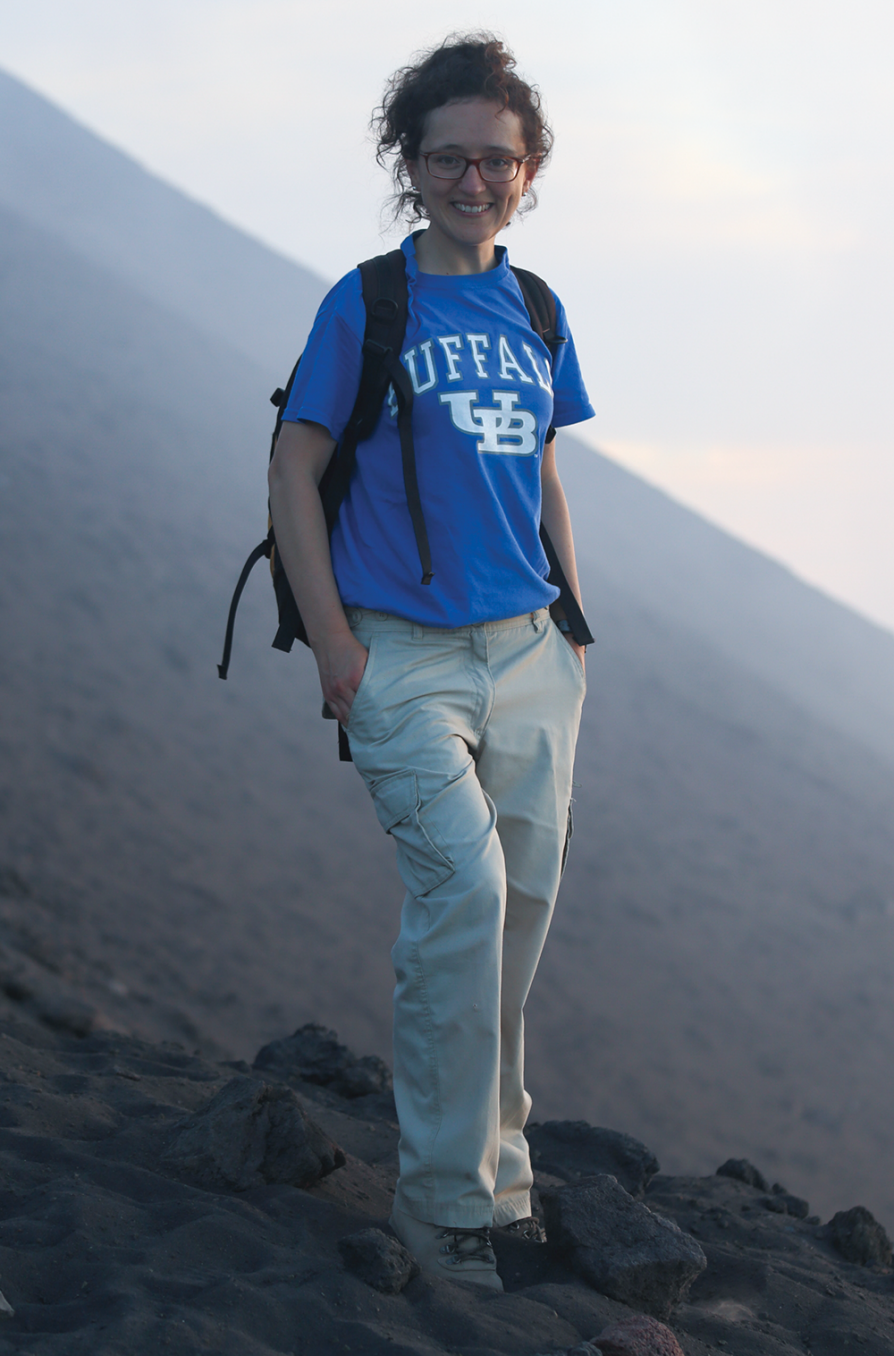
Teresa Ubide stands on Stromboli, a volcano off the coast of Sicily. Credit: Teresa Ubide.

“Even if Jules Verne envisioned it, accessing the inner guts of the volcano physically is impossible so far,” says Teresa Ubide, a volcanologist at the University of Queensland. She has witnessed the beauties and perils of volcanism—both in nearby Indonesia, a volcanically active archipelago that is home to 300 million people, and in her native Spain, which endured the La Palma eruption that lasted for 3 months in 2021 and consumed the town of Todoque.

Ubide has spent years understanding how these volcanoes work and how they are triggered so that we might better predict their eruptions and protect the millions living in their shadow. She takes an approach that Verne might have appreciated: extracting miniscule crystals from cooled magma and turning them into tiny crystal balls. In a 2024 paper in *Nature Geoscience,* Ubide posits that a variety of crystal known as clinopyroxene could serve as “volcanic crystal balls” because of their ability to forecast future eruptions.

“They grow sequentially, like tree rings, and you see changes in the magmatic environment. The rim of the crystal—the last growth ring—is going to record what happens just before the eruption,” Ubide says. “These crystals are the size of a chickpea if you’re very lucky—more often a lentil or grain of salt. The growth zones are microscopic. And you can analyze them with lasers, like we use for eye surgery. So we build this idea of how the plumbing system inside the volcano works, as if opening a doll’s house.”

**Figure:**
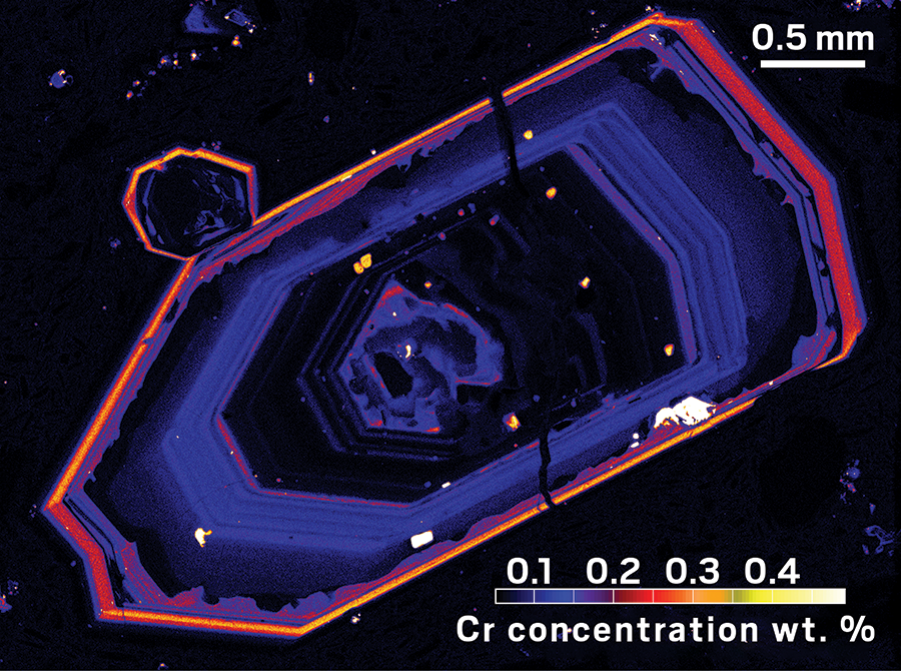
A map of a clinopyroxene crystal, collected from Mount Etna, shows an intense accumulation of chromium (orange) in its outer layer just prior to eruption. Credit: Teresa Ubide.

Ubide’s specialized technique, laser ablation inductively coupled plasma quadrupole mass spectrometry (LA-ICP-MS), differs from more traditional MS. With the traditional approach, researchers must crush their rock sample into a powder and dissolve it in solution for analysis. “The laser allows you to microsample: very tiny parcels, down to a few micrometers,” Ubide says. By shaving away and analyzing each infinitesimal layer, researchers can chart the elemental distribution in 2D, effectively combining all the layers into a visual, millennia-long chemical timeline. Ubide is quick to share images of some of the crystals she has analyzed: vivid, chromatic rainbows of stark, concentric rims, each less than a millimeter wide and representing massive geologic events that occurred lifetimes ago.

Two-dimensional maps of clinopyroxene crystals that passed through a volcano’s magma chamber display a sharp increase in chromium concentration in their outermost rims. This increase indicates the arrival of primitive magma directly from the mantle, which can “tip” a volcanic system to erupt.

Ubide has analyzed clinopyroxene from eruptions as far back as the Roman era. In addition to chronicling a volcano’s history, Ubide’s technique can monitor changes in real time. During the eruption in La Palma in 2021, Ubide lasered the magmatic liquid itself. She found that, about 2 weeks before the end of the volcano’s 85-day path of destruction, the chemical composition changed: chromium oxide concentrations within the clinopyroxene samples leveled off, which suggests that the influx of new magma had begun to slow. In situ monitoring like this may allow communities to better forecast and plan for the length, style, and risks of eruptions.

Ubide’s research on clinopyroxene crystals has also led her to an unexpected application for her method: locating novel sources of copper.

“[Copper]’s such a good conductor of electricity and heat, it's the number 1 thing we need,” Ubide says. “One electric car needs more than 50 kg of copper; one wind turbine needs almost 5 metric tons.” Starting as early as this year, the soaring need for copper—much of it to fuel clean energy technology—is expected to outpace global supply. Demand is forecast to as much as triple within 20 years.

Volcanoes, it turns out, could provide a unique glimpse into the geology of copper formation. “All magmas have a little bit of copper, but it’s very, very rare” that the metal accumulates, Ubide says. To thread that needle, magma that forms deep within the earth must ascend to the surface—surviving a complex gauntlet of geochemical processes, recharging with new injections of magma, and precipitating crystals along the way—until it finally reaches pressures low enough that it can release volatile gases rather than erupting.

If all these stars align, however, the magma heats up sea- or groundwater. That produces a hydrothermal system of geofluids that circulate through the shallow crust and precipitate the increasingly valuable metal into fractured rocks as the fluids cool. “You want the volcano not to erupt because you lose the copper to the atmosphere,” Ubide says.

“The crystals document all these processes,” Ubide explains. But while clinopyroxene proves especially useful for studying why a volcano erupts, it’s not the only crystalline material that doubles as a dutiful geological notary.

Plagioclase, a type of feldspar, is more common than clinopyroxenes in the shallower, silica-rich regions that also produce copper. That means the mineral can help indicate which volcanoes have accumulated the metal. Ubide also uses LA-ICP-MS to analyze this subterranean substance.

As the race for copper intensifies, Ubide and her cohorts have discussed the possibility of outfitting a vehicle to aid in the search for copper deposits. “[If] we get to the point where we can analyze the plagioclase, and if the plagioclase gives you reliable information, you could, in the future, envision a van where you go around the region, get the sample with your hammer, zap the plagioclase, and say, green light, let’s drill here! Or red light, let’s move on,” she says.

While Ubide develops her science, she remains keenly aware of the communities on the receiving end of volcanic hazards that have the most to gain or lose from the coming copper boom. She especially remembers a local guide in Indonesia who shepherded her to the lahar, volcanic mudflow from an eruption decades earlier. “The samples we got there—stunning,” she says.

“And this was all because this local person said, ‘Oh, if you’re interested in crystals, this lahar deposit has lots.’ And he was right. It’s unforgettable,” she recalls. Every time she visits, Ubide draws on knowledge from Indonesian researchers, mine workers, and experienced guides in a multidisciplinary collaboration that is crucial to her work, she says. “We made long-lasting human connections.”

*Jonathan Feakins is a freelance contributor to* Chemical & Engineering News, *an independent news publication of the American Chemical Society.*

